# Link Between Individual Codon Frequencies and Protein Expression: Going Beyond Codon Adaptation Index

**DOI:** 10.3390/ijms252111622

**Published:** 2024-10-29

**Authors:** Konstantin Zaytsev, Natalya Bogatyreva, Alexey Fedorov

**Affiliations:** Bach Institute of Biochemistry, Federal Research Center of Biotechnology of the Russian Academy of Sciences, Moscow 119071, Russia

**Keywords:** gene expression, codon usage, expression prediction, *Escherichia coli*

## Abstract

An important role of a particular synonymous codon composition of a gene in its expression level is well known. There are a number of algorithms optimizing codon usage of recombinant genes to maximize their expression in host cells. Nevertheless, the underlying mechanism remains unsolved and is of significant relevance. In the realm of modern biotechnology, directing protein production to a specific level is crucial for metabolic engineering, genome rewriting and a growing number of other applications. In this study, we propose two new simple statistical and empirical methods for predicting the protein expression level from the nucleotide sequence of the corresponding gene: Codon Expression Index Score (CEIS) and Codon Productivity Score (CPS). Both of these methods are based on the influence of each individual codon in the gene on the overall expression level of the encoded protein and the frequencies of isoacceptors in the species. Our predictions achieve a correlation level of up to r = 0.7 with experimentally measured quantitative proteome data of *Escherichia coli*, which is superior to any previously proposed methods. Our work helps understand how codons determine protein abundances. Based on these methods, it is possible to design proteins optimized for expression in a particular organism.

## 1. Introduction

Most amino acids are represented by two or more codons, and mutations that substitute one codon for a synonymous one do not alter the amino acid in the gene’s final product. Traditionally viewed as silent, these mutations were believed to have minimal impact on phenotype. However, recent studies have uncovered various instances where synonymous mutations play significant roles [1,2,3]. These roles include optimizing gene expression by boosting translation initiation, adjusting translation speed by influencing codon usage and mRNA structures, stabilizing mRNA to prevent premature degradation before translation, and affecting protein folding, degradation, ubiquitination and protein secretion within cells [3]. One of the most impressive experiments showed the effect of synonymous mutations in GFP on a 250-fold change in expression levels [4]. Experiments with TEM-1 β-lactamase suggest that synonymous mutations may have beneficial effects by increasing the expression of an enzyme with low substrate activity [5]. Experiments on *Salmonella enterica* showed that the effects of synonymous mutations are due to the combination of effects on mRNA stability and translation efficiency, which alter the levels of the weak-link enzyme. These studies indicate that synonymous mutations most likely play an essential role, which is currently underestimated [6]. Understanding the mechanism of synonymous mutation impact is important for understanding evolution and variation.

Multiple studies showed that the amount of expressed protein could be increased by replacing the codons in a gene with synonymous ones [7,8,9,10,11]. However, replacing all codons with the preferred ones does not necessarily achieve the maximum protein yield. Instead, it can reduce the balance between codon usage and tRNA abundance, which leads to reduced global translation efficiency [12]. Other possible consequences include changes in protein solubility [13] and incorrect folding [14,15,16].

The ability to change a gene sequence to modify protein expression to the predetermined level is an important industrial and academic challenge. Metabolic engineering often requires the insertion of groups of genes to be expressed with specific individual levels to provide maximum efficiency of the required set of enzymatic reactions while avoiding unnecessary energy cost for production of excessive amounts of proteins [17]. Another trending issue is genome rewriting [18,19,20], which requires changes in synonymous codon sets of protein-encoding genes and may lead to imbalanced production of proteins that harms cell metabolism.

The most popular methods for assessing expression levels are the Codon Adaptation Index (CAI) [21] and tRNA adaptation index (TAI) [22,23,24]. TAI predicts expression levels based on the pool of available tRNAs and the binding efficiency for codons with corresponding isoacceptor tRNAs, showing a prediction accuracy of r = 0.54 on *E. coli* genes [24]. The Codon Adaptation Index (CAI) [21] is the most widely used method for predicting the expression levels from gene sequences. This method is based on determining the organism’s preferred codons for each amino acid from their occurrence in well-expressed genes. The CAI for a gene is calculated as the geometric mean of the frequency of each codon occurrence relative to the most frequent synonymous codon in the training set. The main advantage of the method is that it is very efficient in terms of requiring a fairly small amount of data for training: only a few sequences of well-expressed genes. However, CAI is based on the assumption that there is only one preferred codon for each amino acid, and the higher the proportion of the preferred codons, the higher the level of protein expression. CAI expression prediction accuracy was tested on 96 *E. coli* genes at r = 0.53 level [9].

There are also numerous other indices and methods, which uncover the links between codon usage and different aspects of gene expression [25]. Even though these indices were trained on different data sources, they tend to correlate with each other. One of them is the Relative Codon Bias Score (RCBS) [10]. It measures codon frequency bias by comparing codon frequencies with the frequencies of the individual nucleotides. The authors claimed r = 0.7 expression prediction accuracy after testing on a dataset consisting of 45 *E. coli* genes.

Due to advancements in biotechnology, there are multiple large-scale proteome analysis datasets for different organisms [26,27,28,29,30,31,32]. This allows us to create more powerful methods for expression prediction based on a genome-wide analysis and expand the understanding of expression regulation patterns. The goal of this study was to show the influence each of the individual codons has on the integral level of protein expression. This would allow better optimization for expression of recombinant genes, as well as their alteration to the desired expression level.

## 2. Results

The structure of each gene is unique and depends on the structure and functions of the protein it encodes. To show the differences in the codon structures of the genes coding for highly and lowly expressed proteins, we split the genes from the dataset into four classes based on the levels of the corresponding protein production. The first class contained genes with the highest expression levels, and the fourth class contained genes with the lowest expression levels. We calculated the distributions of codon frequencies for the classes, which allowed us to minimize the individual influence of each gene on the codon distribution while maintaining the factors that influence the overall expression level.

Genes were split into the classes in such a way that each class contained the same number of genes; therefore, the boundaries of the classes have no biological meaning. The expression levels of genes from Class 1 ranged from 356 to 38,022 protein copies per cell, Class 2: from 82 to 356, Class 3: from 18 to 82, Class 4: from 0 to 18.

**Figure 1:**
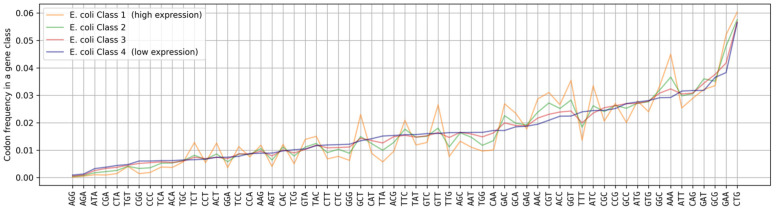
The distribution of codon frequencies in four classes of *E. coli* genes with different levels of protein expression. Codons are sorted by their frequency of occurrence in the class of genes with the lowest expression. The class of genes with the highest levels of expression is shown in orange (Class 1); the class of genes with the lowest expression levels is shown in blue (Class 4); the classes of genes with intermediate expression levels are shown in green (Class 2) and red (Class 3).

Figure 1 shows that for some of the codons, their frequency of occurrence does not depend on the levels of protein expression (for example, AGG, TGT, CCT, CCA, GAG, ATG, CAG, etc.), while for other codons, the differences are significant (for example, TCT, GCT, TTA, GTT, GGT, TTT, AAA, etc.). The frequencies of codon occurrences were calculated as fractions of the 61 possible amino-acid-coding codon occurrences in the genes belonging to a particular class. As can be seen, all differences between codon frequencies are directional and proportional to the differences between the average expression levels of the classes. Therefore, it is obvious that there is a link between the frequency of each individual codon and the integral protein expression level.

**Figure 2:**
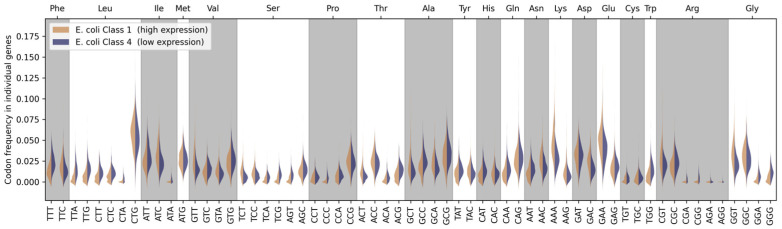
Distributions of the codon appearance frequencies in the individual *E. coli* genes from the class of genes with the highest levels of expression (Class 1), which are shown in orange. Distributions for the class of genes with the lowest expression levels (Class 4) are shown in blue.

Figure 2 shows distributions of codon occurrences in individual genes from Class 1 (highest expression genes) and Class 4 (lowest expression genes). The distributions depend on the number of genes containing such codons and their frequency of appearances in genes. It can be seen that *E. coli* exhibits preferences for some specific codons for several amino acids (Leucine, Isoleucine, Arginine, Glycine), or synonymous codons can be used equally (for example for Phenylalanine). But there is always a difference in codon preferences between genes with different levels of expression. What remains consistent across all codons is that the codon occurrence distribution for genes with low expression levels is more consistent with a normal distribution than for genes with higher expression levels, where there is a higher degree of specificity in the codon frequencies.

**Figure 3:**
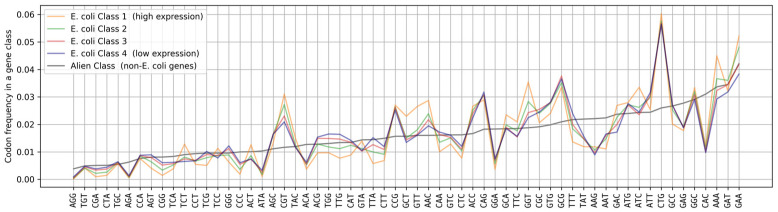
The distribution of codon frequencies in each class of *E. coli* genes and a group of genes from other species (Alien Class). Codons are sorted by the frequency of their appearance in the Alien Class, shown in gray. The class of genes with the highest levels of expression is shown in orange (Class 1); the class of genes with the lowest expression levels is shown in blue (Class 4); the classes of genes with intermediate expression levels are shown in green (Class 2) and red (Class 3).

Figure 3 shows the distribution of codon frequencies in each of the four classes of *E. coli* genes and the Alien Class, consisting of genes derived from other species. We sourced the expression data for these genes from [33]. Among the 1973 alien genes with the highest expression score in the dataset, we randomly chose 422 and combined them into the Alien Class. We can see that the frequencies of some codons are specific to *E. coli* genes, while the frequencies of other codons do not show species specificity. This leads us to assume that codon frequency distributions are specific to particular species, and when used to predict the level of protein expression, it makes sense to take the species-specific nature of the codon distribution into account.

Despite the fact that alien genes were expressed well in *E. coli* in many instances, their codon distributions are quite different from the distributions for native genes. We listed the linear correlation values between distributions for each pair of classes in Table 1. The lowest observed correlation coefficient between a pair of distributions for *E. coli* gene classes is 0.89, while the correlation between *E. coli* classes and the class of alien genes ranges from 0.73 to 0.77. Therefore, we can establish the presence of gene optimization to the specific species in genes with all levels of expression. The absence of such optimization does not necessarily impair gene expression. This also means that the effect of codon usage on the protein expression level is individual for each species.

**Figure 4:**
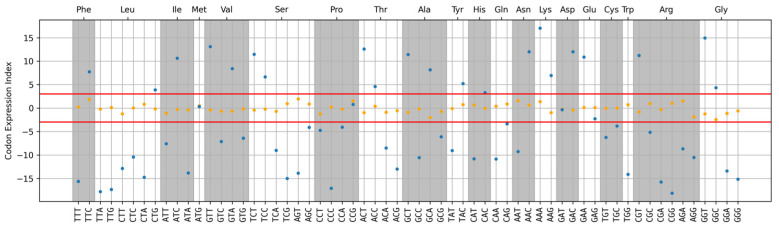
Codon Expression Index (CEI) values show the level of influence of a single codon on the protein expression level of the gene (blue dots). Orange dots represent CEI values calculated for randomly shuffled arrays and are used to determine the boundaries of statistical significance. All random values fall within the range from −3 to 3 (red lines), corresponding to three standard deviations. Therefore, we can assume that codons for which the CEI module is greater than 3 exert a significant effect on the protein expression level.

To analyze the contribution of the individual codons to the integral protein expression level, we introduced the Codon Expression Index (CEI), which shows the level of statistical significance of the correlation between the frequency of each codon occurrence in *E. coli* genes and the expression level of the corresponding protein. Figure 4 shows CEI values for each of the codons. Orange dots represent random CEI values, which we used to determine the statistical significance boundary. All random (orange dots) values fall within the range from −3 to 3 (red lines), corresponding to three standard deviations. Therefore, we can assume that codons for which the CEI module is greater than 3 exert a considerable influence on the integral expression level.

To better understand the process of protein synthesis, we introduced the Codon Productivity (CP) metric. CP shows the average amount of amino acids used by the cell for protein production based on the specific codon, which is defined by codon and corresponding tRNA frequencies.

**Figure 5:**
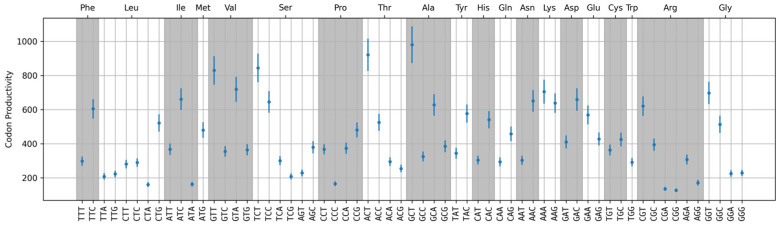
Codon Productivity (CP) values show the contribution of a particular codon to the amount of amino acids utilized during total protein production in a cell. CP values are shown as blue dots. Blue vertical lines show the variability between the three independent experimental expression measurements, corresponding to a single standard deviation.

The dots in Figure 5 represent productivity values for codons. Variability of the values is plotted as a bar for each codon and corresponds to a single standard deviation. Standard deviation was calculated from the variation between three independent experimental measurements. The productivity of most synonymous codons differs by more than the error range; therefore, these differences can be considered statistically significant.

The Codon Productivity (CP) and Codon Expression Index (CEI) values for each codon are listed in the Supplementary Table S1. CP and CEI achieve a very high degree of linear correlation (0.945). That is, both of our proposed metrics can be used interchangeably to analyze the influence of the codons on protein expression. The differences between these methods are due to the different nature of the errors: for CEI, the main factor is the use of rank correlation, and for codon productivity, it is the fact that we calculated it based on a limited set of genes from the organism (the ones for which the expression levels were known).

Interestingly, the length of the gene does not affect its expression level in *E. coli*, unlike higher eukaryotes, for which there is a positive correlation [34]. For *E. coli*, the correlation between the length of a gene and the expression level of the corresponding protein is −0.13, based on the dataset used.

**Figure 6:**
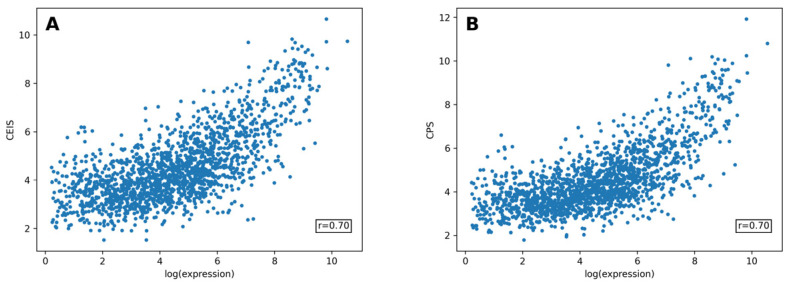
Validation of the expression prediction models on the *E. coli* dataset using 11-fold cross-validation. (**A**). The Codon Expression Index Score (CEIS) is based on the CEI values for the codon’s influence on the integral protein expression. (**B**). The Codon Productivity Score (CPS) is based on the CP values for the average number of amino acids produced based on a single codon. Both models achieve an r = 0.70 linear correlation coefficient between the predicted and actual log expression values.

Both of the proposed metrics—the Codon Expression Index (CEI) and Codon Productivity—were used to predict the expression levels of *E. coli* genes based on their nucleotide sequences. The prediction models were trained and tested using 11-fold cross-validation. Both methods achieved r = 0.70 linear correlation between the predicted and actual expression values (Figure 6).

**Figure 7:**
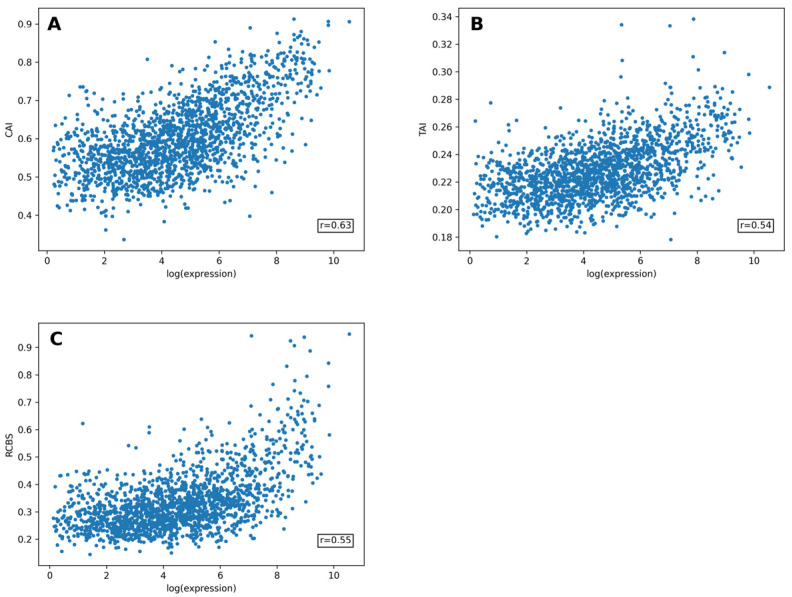
Validation of the models for expression prediction on the *E. coli* dataset. (**A**). The Codon Adaptation Index (CAI) achieves an r = 0.63 linear correlation coefficient with log expression values. (**B**). The tRNA Adaptation Index (TAI) achieves an r = 0.54 linear correlation coefficient with log expression values. (**C**). The Relative Codon Bias Score (RCBS) achieves an r = 0.55 linear correlation coefficient with log expression values.

We compared the prediction accuracy of both models with other existing methods for expression prediction on the same *E. coli* dataset. We tested the Codon Adaptation Index (CAI), tRNA Adaptation Index (TAI) and Relative Codon Bias Score (RCBS) on disjoint training and test sets. The correlation coefficients between the predicted and experimental log values were r = 0.62 for CAI and r = 0.54 for TAI (Figure 7). RCBS [10] had the highest correlation between the predicted and actual expression values, according to the source, at r = 0.70. When tested on the dataset we used, the efficiency of this method turned out to be significantly lower, at r = 0.51.

**Figure 8:**
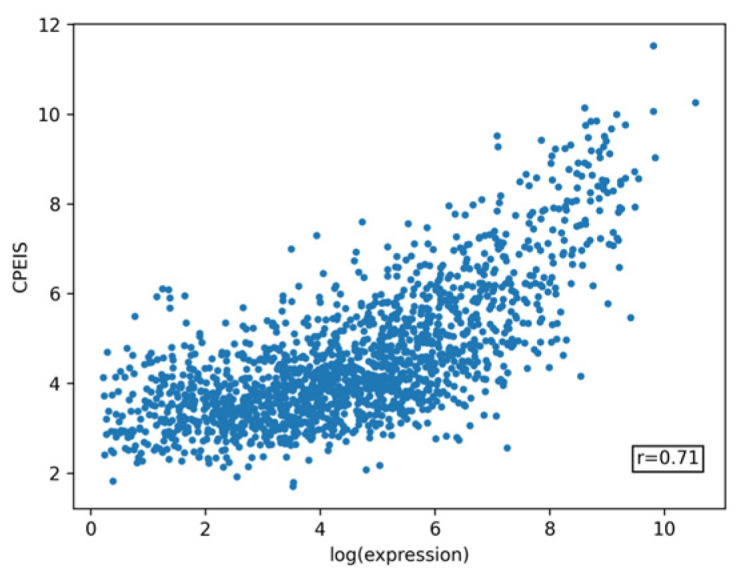
Validation of the expression prediction accuracy for the Codon Pair Expression Index Score (CPEIS) using 11-fold cross-validation. The CPEIS is based on CPEI values for the influence of codon pairs on the integral protein expression. This model achieves an r = 0.71 linear correlation coefficient between the predicted and actual log expression values.

In order to show the influence of correlations between codons on gene expression, we proposed the Codon Pair Expression Index (similar to the CEI but calculated using codon pair frequencies instead of the frequencies of individual codon) and developed an expression prediction model for this index. The CPEI values are listed in the Supplementary Table S2. Pearson’s linear correlation coefficient between the predicted and actual log expression values is r = 0.71 (see Figure 8). The prediction accuracy for CPEIS is moderately higher than for CEIS, while the computational complexity increases significantly, which does not allow us to recommend using this method for expression prediction.

A total of 3721 possible codon pairs do not contain a stop codon. Genes from the dataset used contain all the pairs, except for 14: TCTAGC, CCCCTA, ATTAGG, GTTAGG, TCTAGA, TCTAGG, TCGAGG, CCTAGA, CCTAGG, ACTAGG, GCTAGA, GCTAGG, TATAGG, CGGAGA.

As can be seen in Table 2, for all expression prediction methods, the peak prediction accuracy is achieved for genes with a log expression level above 2 (more than six protein copies per cell). All of the prediction methods presented in the table are based on nucleotide sequence analysis. Therefore, it can be concluded that the regulation of gene expression at low levels is achieved by means of other regulatory processes. At the same time, for genes with higher expression levels, codon frequency is a major regulatory factor. In light of these observations, and the fact that the codon distributions for four classes of *E. coli* genes with different expression levels are highly similar, we suggest that all genes are optimized for a certain organism, regardless of their expression levels.

We created a Python module for calculation of the CEI and CP values and for expression prediction based on these indices. The package is available for download from PyPI as “cei”. The source code for the module is available on Github at “https://github.com/conzaytsev/CodonExpressionIndex”.

## 3. Discussion

Protein expression levels are determined by a variety of factors. These include the promoter strength, transcription rate, ribosome binding site strength, translation rate, degradation rates of both mRNA and protein, as well as the influence of regulatory elements. Misfolding can also affect the efficiency and functionality of the resulting protein, as well as the integral protein level via the protease activity [35]. The presence of all these factors allows for the same expression level to be achieved through numerous distinct combinations [36]. One of the most significant factors affecting protein expression is the frequency of synonymous codon usage [1,37,38,39,40]. Codon usage primarily impacts the speed and efficiency of the translation process (elongation) due to the varying frequencies of the corresponding tRNA molecules. However, several studies have also shown that codon usage can affect the transcription and stability of the mRNA itself [3,41].

We demonstrated that the frequencies of individual codons correlate with the protein expression level in *E. coli*. To capture this relationship, we proposed two new metrics. The first index we called the Codon Expression Index. CEI characterizes the influence of a specific codon on the overall protein expression level. The second index we called Codon Productivity. CP shows the contribution of a particular codon to the amount of amino acids used up during total protein production in a cell. Interestingly, these two quite different methods—the statistical CEI and the empirical CP—showed a very high mutual correlation, with a correlation coefficient of r = 0.945 between their respective values. This suggests that the two metrics can be considered interchangeable, although they have distinct interpretations. The CEI indicates the direction and strength of a particular codon’s influence on the integral level of protein expression. In contrast, the Codon Productivity (CP) values represent the expected number of copies of an amino acid to be used for protein production based on that specific codon.

We used both of the metrics we developed for protein expression prediction and designated the prediction methods as the Codon Expression Index Score (CEIS) and Codon Productivity Score (CPS). When we evaluated the performance of these models, we found that the correlation coefficient between the predicted expression levels of *E. coli* genes and the experimentally measured protein expression levels was 0.7 for both models. This level of correlation represents a significant improvement over all currently widely used predictors. Specifically, our models demonstrated a higher correlation relative to the Codon Adaptation Index (CAI). Other common methods like the tRNA Adaptation Index (TAI) and Relative Codon Bias Score (RCBS) were found to be less effective than CAI in the current dataset. The prediction accuracy was nearly identical between our two models, CEIS and CPS, which is not surprising, given the high correlation we observed previously between the CEI and CP metrics themselves.

Our protein expression prediction models offer several advantages over other popular methods, including greater prediction accuracy. Unlike the widely used Codon Adaptation Index (CAI), our models do not impose artificial limitations, such as restricting each amino acid to the single optimal codon. Instead, CEI calculates the degree of influence each codon has on protein expression, which can be either positive or negative. Additionally, our models can be trained on expression data for any organism under any living condition. We discovered that these methods do not always align with CAI regarding the preferred codons. For example, according to CAI, the ACC codon is the preferred one for threonine because it is the most common in highly expressed genes (Supplementary Table S1). However, both CEI and CP metrics indicate that the ACT codon is more effective at protein production. Similar discrepancies were found for serine, alanine and aspartic acid. This difference arises because, unlike CAI, our methods analyze the dynamics of codon frequency changes among genes with varying expression levels, rather than simply identifying the characteristic patterns of highly expressed genes.

The primary drawback of our models is that they require experimental data on protein expression for the genes of an organism in question. However, we believe that as proteomic data become more widely available for popular species, these models will be able to provide more accurate practical results.

We also computed a range of CPEI values for codon pairs and developed an expression prediction model based on them. The accuracy of this model showed a slight increase compared to the model based on individual codons. It is worth noting that correlations between neighboring codons in *E. coli* have been documented previously [42]. Experimental evidence has also confirmed that correlations between codons can impact protein expression. For example, the use of codon pairs has notably enhanced the expression of synthetic sequences [7], while consecutive CGA codons (arginine) were found to disrupt expression in *E. coli* [43]. We believe that the marginal improvement in prediction accuracy using codon pairs is due to the primarily negative influence of certain codon combinations. These combinations may already be depleted due to the natural optimization of genes during the evolution of a specific organism’s genome. However, considering codon pairs when optimizing gene sequences for recombinant expression purposes could have a significant impact on the protein expression levels.

It is important to note that for the genes exhibiting the lowest expression levels (0–6 gene copies per cell), the correlation between predicted and experimental expression values is considerably below the average. When these genes are excluded from the dataset, the prediction accuracy increases significantly for all considered methods. It is difficult to draw conclusions regarding low-expressed genes because it is challenging to assess the accuracy of data in the low expression range. However, we can assume that genes with the lowest levels of expression are subject to more subtle mechanisms of expression regulation, rather than via codon frequencies. This might be due to the fact that a combination of strong transcription with weak translation is not energy-efficient [36].

Both our methods, CEIS and CPS, achieve a correlation coefficient of r = 0.7 with experimentally measured protein expression levels, which means that almost half of the expression variance can be explained by codon usage. Thus, we clearly conclude that codon usage is a major factor in determining the protein expression level at least in *E. coli.*

We developed our methods based on the experimental data on the expression level of *E. coli* proteins. Therefore, expression prediction using the proposed methods is effective for *E. coli* genes. However, for exogenous sequences (genes from other organisms), the prediction accuracy may become significantly lower. This is because those sequences are not optimized for *E. coli*, and many different expression limitations may arise. Despite this potential decrease in prediction accuracy for exogenous sequences, the influence of codon frequencies on protein expression should remain. Therefore, both CEI and CP indices will likely continue to be relevant and useful for assessing protein expression levels.

The codon distributions for the high- and low-expression *E. coli* genes are more similar to each other than the distributions for genes from other organisms (so-called Alien Class genes in this paper). This suggests that *E. coli* has a species-specific optimization of its gene sequences, regardless of their expression level. The goal of this optimization can be both to achieve the balance between codon usage and tRNA pool ratios and to avoid the formation of toxic mRNAs [44]. The application of our CEI model offers a superior alternative to traditional methods for assessing expression levels within the cellular environment.

## 4. Materials and Methods

### 4.1. Dataset

In this study, we analyzed the protein expression levels for *E. coli* genes. By protein expression, we mean the number of protein copies processed from the corresponding gene, which is a function of all constituent processes, including promoter strength, transcription rate, ribosome binding site strength, translation rate, degradation rates of both mRNA and protein, and so on. The genomic sequence for the *E. coli* strand ATCC 25922 and all the open reading frames (list of genes) were obtained from “https://genomes.atcc.org/genomes/ccbc9e61ad334c2c (accessed on 22 February 2024)”.

The protein abundance data for the *E. coli* strand ATCC 25922 were obtained from the supplementary material in [26]. This dataset is also available at “https://pax-db.org/dataset/511145/3645765292/”. As described in the article, the amount of protein was measured via LC-MS/MS in three repetitions under the same physiological and experimental conditions. To determine the expression level, we used the average of three experimental repetitions in our calculations. The range of non-zero expression values in the dataset varied from 0.06 to 38,022 protein copies per cell. Gene–protein matching was performed using the UniprotID of the encoded proteins. For further analysis, only genes with exactly one single copy in the genome and non-zero expression levels were selected. The final dataset contained 1688 genes.

### 4.2. Gene Clustering

Based on the expression level, we divided genes into four classes: genes with the highest expression levels were combined into Class 1, while those with the lowest levels were combined into Class 4. Genes with expression levels higher than the median and lower than the median were combined into Class 2 and Class 3, respectively. Each of the four classes contained 422 genes.

For each class, we calculated the frequencies of codon appearances for all amino-acid-coding codons. As the gene lengths and the total number of codons differed between the classes, it was necessary to normalize the codon distributions for each class, so that they would sum to 1.

To illustrate the differences between *E. coli* genes and those from other organisms, we created a class comprising non-*E. coli* genes. We used a dataset with genes from different organisms, which were expressed in *E. coli*, obtained from the supplementary material in [33]. A random selection of 422 genes, which exhibited the highest protein production, was extracted from the group of genes that produced the highest amount of protein. These genes were combined into the Alien Class, and the frequencies of codon appearances in the class were also normalized, so that they would sum up to 1.

### 4.3. Codon Expression Index (CEI)

We used Kendall’s rank correlation to determine the influence of each of the codons on the protein expression level [45]. The essence of rank correlation lies in the direction of changes between the values, rather than the specific ratios of values.

For each codon c, except for the stop codons, we created an array of its frequencies in the genes from the dataset $P_{c}=\left\{p_{k}^{c}\right\}$; $k=\left\{1,\ldots ,\;K\right\}$. Here, K is the number of genes in the dataset. This way, we obtained 61 arrays (number of amino-acid-coding codons) with *K* = 1688 values in each one. We also created an array with numbers of protein copies per cell produced by genes from the dataset $E=\left\{e_{k}\right\}$. This array also contained 1688 values. The array indices were unified for all arrays and represented specific genes. We calculated Kendall’s tau between the frequency array for each of the codons and the array of expression values.

(1) $$\tau_{c}=\frac{2}{K\left(K-1\right)}\underset{j<k}{\sum }\left(sgn\left(p_{j}^{c}-p_{k}^{c}\right)sgn\left(e_{j}-e_{k}\right)\right)$$

Here, $\tau_{c}$ is the coefficient of Kendall’s rank correlation.

For each correlation coefficient, we estimated its Z-value [46].

(2) $$Z_{c}=\frac{3\tau_{c}\sqrt{K\left(K-1\right)}}{\sqrt{2\left(2K+5\right)}}$$

$Z_{c}$ shows the number of standard deviations by which the resulting value differs from what would be expected if both arrays were totally independent. Absolute Z-values greater than 3 were considered significant. This enabled us to determine the presence of a positive or negative influence of a given codon frequency on the overall expression. Additionally, the sign of Z-values indicates a positive or negative correlation. To test this, we calculated a set of Z-values for correlations between a codon frequency array and a randomly shuffled expression array. All obtained Z-values fell within the range from −3 to 3, confirming the selected statistical significance threshold. The set of Z-values for codons was designated as the Codon Expression Index (CEI).

### 4.4. Codon Expression Index Score (CEIS)

We used the obtained CEI values to create a protein expression prediction model. We sorted genes from the dataset in the decreasing order of expression and split the dataset into two disjoint sets: a training set and a test set. Each 10 of 11 genes were allocated to the training set, and each 11th gene was allocated to the test set.

CEI values $\left\{Z_{c}\right\}$; $c=\left\{1,\ldots ,\;61\right\}$ were calculated from the training set. Then, for each gene from the training set, we calculated the average CEI value of its codons and combined the results into an array $\left\{C_{k}=\frac{\sum_{i=\left\{1,\ldots ,l\right\}}Z_{c_{i}}}{l}\right\}$; $k=\left\{1,\ldots ,K\right\}$, where K is the number of genes in the training set, and l is the number of codons in a gene. We created a second array containing logarithmic protein expression values. To eliminate negative log values for genes with less than 1 protein copy per cell, we added 1 to each of the expression values before calculating the logarithm: $\log E=\left\{\log\left(e_{k}+1\right)\right\}$. From these two arrays, we calculated linear regression coefficients a and b using the least squares approximation. These coefficients allowed us to scale the predicted values to the actual expression level: $P_{k}=aC_{k}+b$.

Subsequently, the model was subjected to an evaluation on the test set. To enhance the reliability of the prediction accuracy, the dataset was partitioned into training and test sets on 10 additional occasions, ensuring that each gene within the dataset was represented in the test set exactly once. For each pair of sets, we calculated CEI values and regression coefficients from the training set and then predicted the expression values $P_{k}$ for genes from the test set. All the predicted values were combined into an array. Actual expression values $E_{k}$ were combined into a second array. Pearson’s coefficient of linear correlation was employed as a measure of prediction accuracy, calculated between the arrays of predicted and actual expression values.

It is important to note that the use of regression did not affect the prediction accuracy, but it improved the convenience of interpretation of the predicted expression values.

### 4.5. Codon Productivity (CP)

For each codon c, we calculated the total number of the corresponding amino acids used by the cell for protein production.

(3) $$A_{c}=\sum_{k}\left(e_{k}n_{k}^{c}\right)$$

Here, $e_{k}$ is the number of protein copies per cell produced from the gene k. $n_{k}^{c}$ is the number of occurrences of the codon c in the gene k.

Next, for each codon c, we calculated its total number of appearances in the genes from the dataset.

(4) $$N_{c}=\underset{k}{\sum }\left(n_{k}^{c}\right)$$

Codon Productivity (CP) was calculated by dividing the number of amino acid copies corresponding to each codon by the number of codon appearances.

(5) $$CP_{c}=\frac{A_{c}}{N_{c}}$$

Thus, CP shows the average number of amino acids encoded by a particular codon that a cell uses to produce the entire volume of a cell protein.

### 4.6. Codon Productivity Score (CPS)

The model for expression prediction based on CP values is similar to the one for CEI values, except for the use of the average CP for the gene instead of the average CEI.

### 4.7. Codon Adaptation Index (CAI)

For the Codon Adaptation Index (CAI) [21] calculations, we used the CAI v.1.0.5 module for Python [47]. Prediction accuracy was tested on the same data as our methods using 11-fold cross-validation—the same method as with CEIS and CPEIS. Therefore, we were able to compare the prediction accuracy. As a reference set of genes required by CAI, we used a quarter of genes from the training set with the highest expression.

### 4.8. tRNA Adaptation Index (TAI)

For the tRNA Adaptation Index (TAI) [22] calculations, we used the codon bias v.0.3.1 module for Python. The tRNA gene copy numbers we used were acquired from “http://gtrnadb.ucsc.edu/GtRNAdb2/genomes/bacteria/Esch_coli_ATCC_25922/ (accessed on 12 July 2024)”. This method did not require any reference sequences; therefore, we calculated the TAI values for all genes from the dataset and compared them with actual expression values using Pearson’s linear correlation.

### 4.9. Relative Codon Bias Score (RCBS)

For the Relative Codon Bias Score (RCBS) [10] calculations, we also used the codon bias v.0.3.1 module for Python. All parameters were set to default values. This method also did not require any reference sequences; therefore, we calculated prediction accuracy the same way as for TAI.

## 5. Conclusions

We proposed two new indices, showing the effect of the individual codon frequencies on the protein expression level, and we demonstrated that the predictions made using each of these indices correlate with experimentally measured protein abundance values, achieving an r = 0.7 correlation level in *E. coli*. This is superior to any of the traditional methods and indicates that codon frequencies are a major factor in determining protein expression levels, at least in *E. coli*, and can be used for protein expression prediction.

We also showed that the most abundant of the synonymous codons are not necessarily the most effective for protein production. The only downside of the Codon Expression Index is the need for the proteomics data for the species in question. As proteomics data become available for more species, CEI and CP could be used more widely for expression prediction and gene modification to achieve the target protein production levels.

## Figures and Tables

**Table 1:** Coefficients of linear correlation between pairs of codon distributions for four classes of *E. coli* genes, with different levels of protein expression, and the Alien Class, consisting of non-*E. coli* genes.

	Class 1 (High Expression)	Class 2	Class 3	Class 4 (Low Expression)
Class 1 (high expression)	1.00	0.96	0.92	0.89
Class 2	0.96	1.00	0.98	0.97
Class 3	0.92	0.98	1.00	0.99
Class 4 (low expression)	0.89	0.97	0.99	1.00
Class of non-*E. coli* genes	0.73	0.77	0.75	0.73

**Table 2:** Expression prediction accuracy for *E. coli* genes with different levels of protein expression. Prediction accuracy is measured as a coefficient of linear correlation between the predicted and actual log expression values.

			Method					
Log Expression Level	Expression Level (Number of Protein Copies per Cell)	Number of Genes	CEIS	CPEIS	CPS	CAI	TAI	RCBS
0–10	0–38,022	1688	0.702	0.714	0.697	0.625	0.539	0.552
1–10	2–38,022	1624	0.704	0.715	0.702	0.629	0.541	0.564
2–10	6–38,022	1480	0.729	0.742	0.730	0.652	0.546	0.590
3–10	19–38,022	1264	0.717	0.733	0.725	0.631	0.517	0.586
4–10	53–38,022	989	0.704	0.725	0.718	0.602	0.487	0.592

## Data Availability

The genomic sequence for the *E. coli* strand ATCC 25922 and the list of all genes were derived from “https://genomes.atcc.org/genomes/ccbc9e61ad334c2c (accessed on 22 February 2024)”; the expression data for the genes from *E. coli* strand ATCC 25922 were derived from “https://doi.org/10.1016/j.dib.2014.08.004 (accessed on 21 February 2024)”, also available at “https://pax-db.org/dataset/511145/3645765292/”; the “CAI” v.1.0.5 module for Python was derived from Github at “https://github.com/Benjamin-Lee/CodonAdaptationIndex (accessed on 23 March 2024)”; the “codon-bias” v.0.3.1 module for Python was derived from PyPI at “https://pypi.org/project/codon-bias/ (accessed on 28 February 2024)” (it is also available on Zenodo at “https://doi.org/10.5281/zenodo.8039452” or on Github at “https://github.com/alondmnt/codon-bias”); the tRNA gene copy numbers for *E. coli* ATCC 25922 were derived from “http://gtrnadb.ucsc.edu/GtRNAdb2/genomes/bacteria/Esch_coli_ATCC_25922/ (accessed on 12 July 2024)”; the calculation of the Codon Expression Index and Codon Productivity from the proteomics data, as well as the prediction of the gene expression level using these metrics, can be achieved with the “cei” module for Python. This module is available on PyPI at “https://pypi.org/project/cei/”; the source code for the module is available on Github at “https://github.com/conzaytsev/CodonExpressionIndex”.

## Supplementary Materials

The following supporting information can be downloaded at: https://www.mdpi.com/article/10.3390/ijms252111622/s1.

## References

[1] Zhou Z., Dang Y., Zhou M., Li L., Yu C., Fu J., Chen S., Liu Y. (2016). Codon Usage Is an Important Determinant of Gene Expression Levels Largely through Its Effects on Transcription. Proc. Natl. Acad. Sci. USA.

[2] Xu Y., Liu K., Han Y., Xing Y., Zhang Y., Yang Q., Zhou M. (2021). Codon Usage Bias Regulates Gene Expression and Protein Conformation in Yeast Expression System *P. pastoris*. Microb. Cell Fact..

[3] Liu Y., Yang Q., Zhao F. (2021). Synonymous but Not Silent: The Codon Usage Code for Gene Expression and Protein Folding. Annu. Rev. Biochem..

[4] Kudla G., Murray A.W., Tollervey D., Plotkin J.B. (2009). Coding-Sequence Determinants of Gene Expression in *Escherichia coli*. Science.

[5] Zwart M.P., Schenk M.F., Hwang S., Koopmanschap B., de Lange N., van de Pol L., Nga T.T.T., Szendro I.G., Krug J., de Visser J.A.G.M. (2018). Unraveling the Causes of Adaptive Benefits of Synonymous Mutations in TEM-1 β-Lactamase. Heredity.

[6] Dhindsa R.S., Copeland B.R., Mustoe A.M., Goldstein D.B. (2020). Natural Selection Shapes Codon Usage in the Human Genome. Am. J. Hum. Genet..

[7] Huang Y., Lin T., Lu L., Cai F., Lin J., Jiang Y., Lin Y. (2021). Codon Pair Optimization (CPO): A Software Tool for Synthetic Gene Design Based on Codon Pair Bias to Improve the Expression of Recombinant Proteins in Pichia Pastoris. Microb. Cell Fact..

[8] Welch M., Govindarajan S., Ness J.E., Villalobos A., Gurney A., Minshull J., Gustafsson C. (2009). Design Parameters to Control Synthetic Gene Expression in *Escherichia coli*. PLoS ONE.

[9] Henry I., Sharp P.M. (2007). Predicting Gene Expression Level from Codon Usage Bias. Mol. Biol. Evol..

[10] Roymondal U., Das S., Sahoo S. (2009). Predicting Gene Expression Level from Relative Codon Usage Bias: An Application to *Escherichia coli* Genome. DNA Res..

[11] Ding Z., Guan F., Xu G., Wang Y., Yan Y., Zhang W., Wu N., Yao B., Huang H., Tuller T. (2022). MPEPE, a Predictive Approach to Improve Protein Expression in *E. coli* Based on Deep Learning. Comput. Struct. Biotechnol. J..

[12] Frumkin I., Lajoie M.J., Gregg C.J., Hornung G., Church G.M., Pilpel Y. (2018). Codon Usage of Highly Expressed Genes Affects Proteome-Wide Translation Efficiency. Proc. Natl. Acad. Sci. USA.

[13] Hurley J.M., Dunlap J.C. (2013). A Fable of Too Much Too Fast. Nature.

[14] Komar A.A., Lesnik T., Reiss C. (1999). Synonymous Codon Substitutions Affect Ribosome Traffic and Protein Folding during in Vitro Translation. FEBS Lett..

[15] Cortazzo P., Cerveñansky C., Marín M., Reiss C., Ehrlich R., Deana A. (2002). Silent Mutations Affect in Vivo Protein Folding in *Escherichia coli*. Biochem. Biophys. Res. Commun..

[16] Liu Y. (2020). A Code within the Genetic Code: Codon Usage Regulates Co-Translational Protein Folding. Cell Commun. Signal..

[17] Kafri M., Metzl-Raz E., Jona G., Barkai N. (2016). The Cost of Protein Production. Cell Rep..

[18] Annaluru N., Muller H., Mitchell L.A., Ramalingam S., Stracquadanio G., Richardson S.M., Dymond J.S., Kuang Z., Scheifele L.Z., Cooper E.M. (2014). Total Synthesis of a Functional Designer Eukaryotic Chromosome. Science.

[19] Hutchison C.A., Chuang R.-Y., Noskov V.N., Assad-Garcia N., Deerinck T.J., Ellisman M.H., Gill J., Kannan K., Karas B.J., Ma L. (2016). Design and Synthesis of a Minimal Bacterial Genome. Science.

[20] Venetz J.E., Del Medico L., Wölfle A., Schächle P., Bucher Y., Appert D., Tschan F., Flores-Tinoco C.E., van Kooten M., Guennoun R. (2019). Chemical Synthesis Rewriting of a Bacterial Genome to Achieve Design Flexibility and Biological Functionality. Proc. Natl. Acad. Sci. USA.

[21] Sharp P.M., Li W.-H. (1987). The Codon Adaptation Index-a Measure of Directional Synonymous Codon Usage Bias, and Its Potential Applications. Nucleic Acids Res..

[22] Reis M.D., Savva R., Wernisch L. (2004). Solving the Riddle of Codon Usage Preferences: A Test for Translational Selection. Nucleic Acids Res..

[23] Sabi R., Volvovitch Daniel R., Tuller T. (2017). StAIcalc: TRNA Adaptation Index Calculator Based on Species-Specific Weights. Bioinformatics.

[24] Anwar A.M., Khodary S.M., Ahmed E.A., Osama A., Ezzeldin S., Tanios A., Mahgoub S., Magdeldin S. (2023). GtAI: An Improved Species-Specific TRNA Adaptation Index Using the Genetic Algorithm. Front. Mol. Biosci..

[25] Bahiri-Elitzur S., Tuller T. (2021). Codon-Based Indices for Modeling Gene Expression and Transcript Evolution. Comput. Struct. Biotechnol. J..

[26] Wiśniewski J.R., Rakus D. (2014). Quantitative Analysis of the *Escherichia coli* Proteome. Data Br..

[27] Schmidt A., Kochanowski K., Vedelaar S., Ahrné E., Volkmer B., Callipo L., Knoops K., Bauer M., Aebersold R., Heinemann M. (2016). The Quantitative and Condition-Dependent *Escherichia coli* Proteome. Nat. Biotechnol..

[28] Mateus A., Bobonis J., Kurzawa N., Stein F., Helm D., Hevler J., Typas A., Savitski M.M. (2018). Thermal Proteome Profiling in Bacteria: Probing Protein State in Vivo. Mol. Syst. Biol..

[29] Lawless C., Holman S.W., Brownridge P., Lanthaler K., Harman V.M., Watkins R., Hammond D.E., Miller R.L., Sims P.F.G., Grant C.M. (2016). Direct and Absolute Quantification of over 1800 Yeast Proteins via Selected Reaction Monitoring. Mol. Cell. Proteom..

[30] Lahtvee P.-J., Sánchez B.J., Smialowska A., Kasvandik S., Elsemman I.E., Gatto F., Nielsen J. (2017). Absolute Quantification of Protein and MRNA Abundances Demonstrate Variability in Gene-Specific Translation Efficiency in Yeast. Cell Syst..

[31] Ho B., Baryshnikova A., Brown G.W. (2018). Unification of Protein Abundance Datasets Yields a Quantitative Saccharomyces Cerevisiae Proteome. Cell Syst..

[32] Huang Q., Szklarczyk D., Wang M., Simonovic M., von Mering C. (2023). PaxDb 5.0: Curated Protein Quantification Data Suggests Adaptive Proteome Changes in Yeasts. Mol. Cell. Proteom..

[33] Boël G., Letso R., Neely H., Price W.N., Wong K.-H., Su M., Luff J.D., Valecha M., Everett J.K., Acton T.B. (2016). Codon Influence on Protein Expression in *E. coli* Correlates with MRNA Levels. Nature.

[34] Grishkevich V., Yanai I. (2014). Gene Length and Expression Level Shape Genomic Novelties. Genome Res..

[35] Gur E., Sauer R.T. (2008). Recognition of Misfolded Proteins by Lon, a AAA + Protease. Genes Dev..

[36] Hausser J., Mayo A., Keren L., Alon U. (2019). Central Dogma Rates and the Trade-off between Precision and Economy in Gene Expression. Nat. Commun..

[37] Presnyak V., Alhusaini N., Chen Y.-H., Martin S., Morris N., Kline N., Olson S., Weinberg D., Baker K.E., Graveley B.R. (2015). Codon Optimality Is a Major Determinant of MRNA Stability. Cell.

[38] Yu C.-H., Dang Y., Zhou Z., Wu C., Zhao F., Sachs M.S., Liu Y. (2015). Codon Usage Influences the Local Rate of Translation Elongation to Regulate Co-Translational Protein Folding. Mol. Cell.

[39] Yang Q., Yu C.-H., Zhao F., Dang Y., Wu C., Xie P., Sachs M.S., Liu Y. (2019). ERF1 Mediates Codon Usage Effects on MRNA Translation Efficiency through Premature Termination at Rare Codons. Nucleic Acids Res..

[40] Plotkin J.B., Kudla G. (2011). Synonymous but Not the Same: The Causes and Consequences of Codon Bias. Nat. Rev. Genet..

[41] Zhao F., Zhou Z., Dang Y., Na H., Adam C., Lipzen A., Ng V., Grigoriev I.V., Liu Y. (2021). Genome-Wide Role of Codon Usage on Transcription and Identification of Potential Regulators. Proc. Natl. Acad. Sci. USA.

[42] Gutman G.A., Hatfield G.W. (1989). Nonrandom Utilization of Codon Pairs in *Escherichia coli*. Proc. Natl. Acad. Sci. USA.

[43] Curran J.F. (1995). Decoding with the A:I Wobble Pair Is Inefficient. Nucleic Acids Res..

[44] Mittal P., Brindle J., Stephen J., Plotkin J.B., Kudla G. (2018). Codon Usage Influences Fitness through RNA Toxicity. Proc. Natl. Acad. Sci. USA.

[45] Kendall M.G. (1938). A New Measure of Rank Correlation. Biometrika.

[46] Hoeffding W. (1948). A Class of Statistics with Asymptotically Normal Distribution. Ann. Math. Stat..

[47] Lee B.D. (2018). Python Implementation of Codon Adaptation Index. J. Open Source Softw..

