# Supplementary material for: Link Between Individual Codon Frequencies and Protein Expression: Going Beyond Codon Adaptation Index
Source: Int J Mol Sci. 2024 Oct 29;25(21):11622. doi: 10.3390/ijms252111622 (PMC11546221; doi:10.3390/ijms252111622)
Supplement: Supplementary file 1 [file ijms-25-11622-s001.zip › Supplementary/Supplementary Table S1.pdf]

CEI and CP Summary Table.

“AA copies per codon” column is the total amount of a certain amino acid produced by the cell from the corresponding codon.
“Codon copies in dataset” column is the total number of a certain codon in the *E. coli* dataset.
“Codon productivity” is calculated as a ratio of “AA copies per codon” to “Codon copies in the dataset”.
“Codon copies in Class 1” is the total number of each of the codons in the class of *E. coli* genes with the highest expression numbers. It is used by the Codon adaptation index to determine the preferred codons.
“Codon expression index” is a measure of codon influence on the integral protein expression level of a gene.

AA	Codon	AA copies per codon	Codon copies in dataset	Codon copies in Class 1	Codon expression index	Codon productivity
Phe	TTT	3392215	11371	1808	-15,62	298±27
	TTC	6156987	10182	2774	7,8	605±57
Leu	TTA	1372317	6571	761	-17,81	209±19
	TTG	1674723	7506	1021	-17,36	223±19
	CTT	1627308	5794	905	-12,85	281±25
	CTC	1777978	6128	1029	-10,44	290±25
	CTA	299309	1856	194	-14,76	161±14
	CTG	17750260	34032	8005	3,86	522±51
Ile	ATT	6405566	17394	3370	-7,6	368±34
	ATC	10410008	15740	4445	10,6	661±64
	ATA	215098	1318	138	-13,84	163±13
Met	ATG	7773239	16178	3708	0,33	480±46
Val	GTT	9306907	11213	3522	13,08	830±84
	GTC	3121197	8786	1704	-7,12	355±31
	GTA	4814578	6696	1848	8,43	719±73
	GTG	5817561	15941	3188	-6,42	365±33
Ser	TCT	4262417	5046	1699	11,46	845±84

AA	Codon	AA copies per codon	Codon copies in dataset	Codon copies in Class 1	Codon expression index	Codon productivity
**Phe**	TTT	3392215	11371	1808	-15,62	298±27
	TTC	6156987	10182	2774	7,8	605±57
	TCC	3429798	5314	1501	6,7	645±63
	TCA	943551	3138	512	-9,00	301±27
	TCG	1005401	4817	671	-15,01	209±18
	AGT	940612	4108	539	-13,85	229±20
	AGC	3519700	9275	1761	-4,10	379±36
**Pro**	CCT	1402942	3817	724	-4,73	368±30
	CCC	427069	2572	249	-17,10	166±14
	CCA	1849491	4943	1003	-4,07	374±33
	CCG	7419731	15424	3588	0,78	481±44
**Thr**	ACT	4829225	5237	1672	12,60	922±95
	ACC	7567292	14407	3532	4,62	525±50
	ACA	915339	3101	492	-8,51	295±25
	ACG	2010523	7877	1278	-12,97	255±22
**Ala**	GCT	9368522	9552	3050	11,44	981±108
	GCC	4806426	14765	2668	-10,54	326±29
	GCA	7435500	11844	3118	8,18	628±63
	GCG	8145107	21120	4449	-6,14	386±35
**Tyr**	TAT	2926522	8484	1579	-9,05	345±32
	TAC	4311932	7473	1998	5,23	577±53
**His**	CAT	2218642	7301	1175	-10,82	304±25
	CAC	3420104	6321	1612	3,30	541±51
**Gin**	CAA	2506994	8534	1337	-10,86	294±27
	CAG	8285246	18063	3844	-3,4	459±43
**Asn**	AAT	2628454	8665	1468	-9,25	303±27

AA	Codon	AA copies per codon	Codon copies in dataset	Codon copies in Class 1	Codon expression index	Codon productivity
Phe	TTT	3392215	11371	1808	-15,62	298±27
	TTC	6156987	10182	2774	7,8	605±57
	AAC	8912002	13684	3810	12,01	651±64
Lys	AAA	14714089	20869	5970	17,04	705±71
	AAG	3823969	5993	1569	6,93	638±57
Asp	GAT	8146115	19817	4270	-0,35	411±38
	GAC	8319457	12620	3570	11,99	659±66
Glu	GAA	15004733	26349	6918	10,87	569±55
	GAG	4715266	11015	2355	-2,26	428±39
Cys	TGT	942800	2593	545	-6,2	364±32
	TGC	1536165	3612	763	-3,82	425±40
Trp	TGG	2308168	7906	1285	-14,12	292±25
Arg	CGT	9257924	14914	4119	11,22	621±57
	CGC	5535695	14029	2737	-5,14	395±36
	CGA	211629	1564	126	-15,77	135±12
	CGG	311218	2438	192	-18,14	128±11
	AGA	164818	536	68	-8,7	307±29
	AGG	54611	318	23	-10,5	172±16
Gly	GGT	11223904	16081	4703	14,96	698±65
	GGC	9459232	18401	4428	4,35	514±50
	GGA	805090	3561	482	-13,39	226±19
	GGG	1324437	5776	833	-15,15	229±19
